# Metabolic associated fatty liver disease (MAFLD): assessing the knowledge and practice of primary care doctors in Seremban District, Negeri Sembilan

**DOI:** 10.51866/oa.629

**Published:** 2024-08-16

**Authors:** Kalaivaani Vijan, Athirah Ali, Nur Adhajirin Mohamed Idrus, Priscilla Lourdesamy, Shamini Margammuthu, Suguna Perumal, Cheong Lieng Teng, Imran Ahmad

**Affiliations:** 1 MBBS, icFRACGP, Klinik Kesihatan Kuala Pilah Jalan Macpherson, Kampung Tebat Kening, Kuala Pilah, Negeri Sembilan, Malaysia. Email: kalai88_libra@hotmail.com; 2 MD, icFRACGP, Klinik Kesihatan Salak, Jalan Salak, Sepang, Selangor, Malaysia.; 3 MD, Klinik Kesihatan Mantin, Jalan Besar Mantin, Mantin, Negeri Sembilan, Malaysia.; 4 MBBS, Klinik Kesihatan Sikamat, Jalan Tunku Kurshiah Atas, Seremban, Negeri Sembilan, Malaysia.; 5 MD, Klinik Kesihatan Jelebu, Kuala Klawang, Jelebu, Negeri Sembilan, Malaysia.; 6 Klinik Kesihatan Port Dickson, Jalan Seremban Kampung Dhobi, Port Dickson, Negeri Sembilan, Malaysia.; 7 MBBS, M. Family Medicine, Department of Family Medicine, International Medical University, 126, Jalan Jalil Perkasa 19, Bukit, Jalil, Kuala Lumpur, Malaysia.; 8 MBBS, M. Family Medicine, Department of Family Medicine, Universiti Sains Malaysia, Kampus, Kesihatan, Jalan Raja Perempuan, Zainab 2, Kota Bharu, Kelantan, Malaysia.

**Keywords:** Fatty liver, Obesity, Metabolic syndrome, Malaysia, Primary care physician

## Abstract

**Introduction:**

Metabolic-associated fatty liver disease (MAFLD) is the liver manifestation of metabolic syndrome, which is commonly seen in primary care settings. This study aimed to determine the knowledge and practice of primary care physicians regarding MAFLD in Seremban District, Negeri Sembilan.

**Methods:**

This cross-sectional study was conducted among medical officers in 14 health clinics in Seremban District, using a validated, self-administered online questionnaire.

**Results:**

A total of 240 medical officers from 14 health clinics in Seremban District, participated in this study. Most participants (85.4%) passed the knowledge test. Their practice was acceptable, but only a minority were familiar with non-invasive testing of liver fibrosis (e.g. APRI or FIB-4), medication and specific diet for the treatment of MAFLD.

**Conclusion:**

Most primary care physicians in Seremban District are knowledgeable in identifying risk factors and managing patients with MAFLD. However, there are still areas to improve in terms of management, particularly regarding the use of silymarin, vitamin E and pioglitazone.

## Introduction

Metabolic-associated fatty liver disease (MAFLD), which was previously called non-alcoholic fatty liver disease (NAFLD), is the most common chronic liver disease worldwide, affecting about a quarter of the global population.^1^ It is closely associated with metabolic disorders such as obesity, type 2 diabetes mellitus (DM) and dyslipidaemia, which are commonly seen in health clinics. Other identified risk factors such as hypothyroidism, polycystic ovarian syndrome, obstructive sleep apnoea, hypopituitarism and hypogonadism have been described in Western countries, but these associations are yet to be investigated adequately in the Asia Pacific region.^2^

MAFLD is a spectrum of conditions ranging from fatty infiltration of the liver to steatohepatitis, fibrosis and cirrhosis. It involves histologically like changes seen in alcoholic liver disease but without a history of significant amounts of alcohol intake.^3^ If left untreated, MAFLD can progress to more serious liver diseases such as cirrhosis and liver cancer.

In 2020, a group of international experts reached a consensus to have a comprehensive and simple term that is independent of other liver diseases known as ‘metabolic (dysfUnction)- associated fatty liver disease’ and introduced a simple set of “positive” diagnostic criteria.^4^ While cardiovascular disease is the leading cause of mortality, patients with MAFLD with more severe liver disease are at an increased risk of liver-related complications and mortality. Previous studies on the knowledge of MAFLD in other countries have shown that a significant proportion of doctors do not have sufficient knowledge on how to diagnose and treat the disease.^5-7^

Currently, MAFLD is a growing health concern in Malaysia with a prevalence that is comparable to that overseas; 22.7% of individuals are detected to have MAFLD via health checkups^8^ and 49.6% among patients with DM.^9-10^ A study conducted in Hong Kong showed that the prevalence of NAFLD-related cirrhosis among patients with type 2 DM diagnosed via transient elastography was 11.2%.^11^ The worldwide estimated incidence of hepatocellular carcinoma in patients with NAFLD-related cirrhosis ranges from 0.5% to 2.6% according to Mattos et al.^12^ Primary care doctors should have better knowledge regarding MAFLD so that they can detect it early, manage it accordingly and are aware when to refer to a gastroenterologist for shared care.

Limited studies have evaluated MAFLD awareness among primary care physicians, and no studies have been performed locally in Malaysia.^13^ We hypothesised that a significant knowledge gap exists among primary care physicians regarding the diagnosis and management of MAFLD. Hence, this study aimed to assess the knowledge level and determine the practice patterns regarding MAFLD among primary care physicians in health clinics in Seremban District to ensure a better quality of healthcare.

## Methods

### Study design, setting and participants

This quantitative study adopted a cross-sectional design and involved medical officers in health clinics in Seremban District, Negeri Sembilan. An online survey was conducted in all health clinics under Seremban District, as an online method was deemed more convenient for collecting and analysing data. All 14 health clinics in the district were sampled via universal sampling. A total of 240 medical officers voluntarily responded, yielding a response rate of 84.5%. Non-specialist primary care doctors who were providing clinical care and were working in the 14 health clinics in Seremban District from September to October 2022 were included. Doctors who accomplished only public health or administrative duties were excluded.

A questionnaire was sent to the respective medical officers-in-charge of each clinic via WhatsApp; these officers were tasked to distribute the questionnaire to only medical officers in their health clinics to ensure that the study population was sampled accurately.

### Study instrument

The questionnaire was developed by our team based on similar studies conducted by Matthias et al and Younossi et al and an international guideline.^5-7^ It was created in the English language and consisted of three sections with a total of 11 questions. The first section assessed the participants’ sociodemographic and practice details such as age, sex, place of practice, unit of practice, ongoing postgraduate training (GCFM/ATFM/MInTFM/Masters) and years of service in primary care. The second section of the questionnaire measured the knowledge regarding MAFLD including risk factors, screening, methods of diagnosis, management options, progression and complications of MAFLD, while the third section evaluated practice. The answers to the questionnaire were categorised based on the chosen single best answer or statement with categories *(yeslnolnot sure).*

The instrument underwent face and content validity testing by a panel of experts in MAFLD comprising academic family medicine specialists (FMSs) and gastroenterologists from the International Medical University and Universiti Sains Malaysia. The content of the questionnaire was checked by the panel.

The questionnaire links were distributed via WhatsApp to the medical officers-in-charge of the respective clinics. The questionnaire was answered via Google Forms, which were linked to the email accounts of participants. This ensured that each participant could submit only a single response.

In analysing the knowledge score, a passing mark of 57% was set, which was calculated using 60% of the highest score according to Cohen’s method.^14^ During standard setting, a criterion-referenced method is usually used to set the passing mark. In the actual study, the knowledge score of participants turned out substantially better than originally perceived by the standard-setting expert panel. In some criterion-reference standard settings, a substantially high failure rate may be reported,^14^ but our study showed the opposite. We chose this method, as Cohen-Schotanus and van der Vleuten^14^ suggested that a combination of norm- and criterion-reference methods may be better since it considers a pre-fixed cut-off score (criterion reference: 60%) and a relative point of reference (norm reference highest score or 95th percentile). Cohen’s method reduces the disadvantages of both criterion-and norm- referenced standards (e.g. highly variable cut-off scores and failure rates) and is considered more acceptable and affordable.^14^ The questionnaire is available upon request to the corresponding author.

A pilot study was conducted on 21 medical officers at Klinik Kesihatan Salak, Selangor, before dissemination to our study population in Seremban District, Negeri Sembilan. Reliability testing of the questionnaire was conducted based on the pilot study data, which yielded a Cronbach’s *a* of 0.7, indicating that the questionnaire was reliable.

### Data analysis

Data were analysed using IBM SPSS for Windows, version 26 (IBM Corp, Armonk, New York, USA) Categorical variables were compared using the chi-square test. Continuous variables were summarised as means or medians, as appropriate. Linear correlation was analysed using Spearman’s correlation coefficients. The statistical significance level was set at P<0.05.

## Results

### Demographic data

Of 284 medical officers who were sent the study questionnaire, 240 responded (response rate: 84.5%). The majority of the participants were women (85.8%). The mean age was 35 years (standard deviation: ±5.53). Forty-six medical officers were undergoing postgraduate training in family medicine (19.2%). Around 60% had served in primary care for >5 years. Most participants were working in outpatient departments at the time of questionnaire completion. The demographic and practice data of the participants are further detailed in Table 1.

### Knowledge score

The knowledge scores of the participants are shown in Table 2 with details of the knowledge questions shown in Table 3. The median knowledge score was 72.22 (range: 33.33-94.44, interquartile range: 16.67). The knowledge score had a skewness of -0.45; hence, the measure of central tendency was reported in medians and interquartile ranges. A total of 205 participants passed the knowledge test (85.4%). The passing rate was higher among the participants with postgraduate training than among those without (97.8% vs 91.2%, P=0.127). We analysed the factors affecting the passing rate as shown in Table 5 (current unit of practice, age, years of service, postgraduate training and sex), but the findings were not statistically significant. However, postgraduate training and the actual knowledge score yielded significant findings (Mann-Whitney U test, P<0.001). There was a significant linear correlation between age and the knowledge score (Spearman’s p=0.183, P=0.004).

**Table 1:** Demographic and practice data

Characteristics	Number (%)
***Sex***	
Male	34 (14.2)
Female	206 (85.8)
***Age, year***	
<30	33 (13.8)
30-39	176 (73.3)
40-49	22 (9.2)
50-59	9 (3.8)
***Educational level***	
Postgraduate trainee	46 (19.2)
Non-postgraduate trainee	194 (80.8)
***Current unit of practice***	
OPD/fever/CAC/school	146 (60.8)
MCH	46 (19.2)
NCD	48 (20.0)
***Years of service in primary care***	
<5	96 (40.0)
≥5	144 (60.0)

OPD, outpatient department; fever, fever clinic; CAC, COVID assessment centre; school, school health team; MCH, maternal and child health; NCD, non-communicable disease.

**Table 2:** Knowledge score (passing rate) between the postgraduate and non-postgraduate trainees.

	Postgraduate trainees	Non-postgraduate trainees
Pass	45 (97.8)	177 (91.2)
Fail	1 (2.2)	17 (8.8)
Total	46	194

### Responses to the practice questions

We analysed the responses to the practice questions as frequencies (depicted in Table 4) as there were no local guidelines to set the best practice. In screening MAFLD, 67.9% of the participants indicated screening patients aged 40 years and above; 95.4%, patients with DM; 67.5%, patients with hypertension; and 98.3%, patients who are overweight or obese. When suspecting a diagnosis of MAFLD, 99.6% would request a liver function test (LFT); 97.9% would request an ultrasound of the liver; 47.1% would calculate the APRI score; 44.2% would calculate the FIB-4 score; and 54.2% would calculate the fatty liver index.

Regarding the management options for MAFLD, the majority of the participants agreed on weight reduction (98.3%). The use of pioglitazone was chosen by only 30% of the participants.

Approximately 61.7% thought that a hypocaloric diet would help manage MAFLD, and 57.5% agreed that metformin would help.

Most participants (67.9%) would refer to gastroenterologists when the liver stiffness test result is positive. Only 36.7% of the participants would refer on a patient request basis; 47.5% would refer as soon as a diagnosis of MAFLD is suggestive; and 65.8% would refer to a gastroenterologist when their patients with MAFLD have two or more comorbidities.

**Table 3:** Knowledge regarding MAFLD.

	Answer (correct or incorrect)	Number (%) of respondents answering correctly
***The following conditions are associated with MAFLD:***		
Sedentary lifestyle	Correct	202 (84.2)
Hyperthyroidism	Incorrect	136 (56.7)
Diabetes mellitus	Correct	230 (95.8)
Obesity	Correct	239 (99.6)
Insulin resistance	Correct	216 (90.0)
***Patients with MAFLD in primary care settings are usually asymptomatic.***	Correct	218 (90.8)
***MAFLD can occur in adults with a BMI of <23 kg/m^2^.***	Correct	104 (43.3)
***The following laboratory results occur more commonly in MAFLD:***		
High platelet count	Incorrect	140 (58.3)
AST/ALT ratio of <1	Correct	110 (45.8)
High triglyceride level	Correct	227 (94.6)
High HDL-cholesterol level	Incorrect	151 (62.9)
High gamma-glutamyl transferase level	Correct	159 (66.3)
***Gold standard method for the diagnosis offatty liver***		
Liver biopsy	Correct	96 (40.0)
***MAFLD can increase the risk for the following:***		
Cardiovascular disease	Correct	207 (86.3)
Diabetes mellitus	Correct	182 (75.8)
Liver cirrhosis	Correct	220 (91.7)
Hepatocellular carcinoma	Correct	183 (76.3)
***Milk thistle (silymarin) can prevent the progression of MAFLD.***	Incorrect	30 (12.5)

**Table 4:** Responses to the practice questions.

	Yes	No	Not sure
***Categories of patients that the participants would screen for MAFLD***			
Age of >40 years	163 (67.9)	65 (27.1)	12 (5.0)
Diabetes mellitus	229 (95.4)	8 (3.3)	3 (1.3)
Hypertension	162 (67.5)	64 (26.7)	14 (5.8)
Obesity/overweight	236 (98.3)	2 (0.8)	2 (0.8)
***Investigation the participants would order if they clinically suspect MAFLD***			
Liver function test	239 (99.6)	1 (0.4)	0 (0)
Liver ultrasound	235 (97.9)	4 (1.7)	1 (0.4)
APRI (AST-to-platelet ratio index) score	113 (47.1)	49 (20.4)	78 (32.5)
Fibrosis-4 score	106 (44.2)	42 (17.5)	92 (38.3)
Fatty liver index	130 (54.2)	30 (12.5)	80 (33.3)
***Management of MAFLD***			
Aerobic exercise	229 (95.4)	6 (2.5)	5 (2.1)
Vitamin E	78 (32.5)	78 (32.5)	84 (35.0)
Metformin	138 (57.5)	57 (23.8)	45 (18.8)
Pioglitazone	68 (28.3)	63 (26.3)	109 (45.4)
Weight reduction	236 (98.3)	2 (0.8)	2 (0.8)
Hypocaloric diet	148 (61.7)	39 (16.3)	53 (22.1)
Ketogenic diet	72 (30.0)	84 (35.0)	84 (35.0)
Mediterranean diet	84 (35.0)	56 (23.3)	100 (41.7)
***Indication for gastroenterologist referral***			
As soon as the investigations are suggestive of MAFLD	114 (47.5)	103 (42.9)	23 (9.6)
Presence of two or more comorbidities	158 (65.8)	50 (20.8)	32 (13.3)
Patient request	88 (36.7)	123 (51.2)	29 (12.1)
Liver stiffness positivity	163 (67.9)	14 (5.8)	63 (26.3)

**Table 5:** Summary of the factors affecting the passing rate (pass/fail).

Factors	P-value
Current unit of practice	0.622
Age	0.932
Years of service (≥5 vs <5)	0.689
Postgraduate training	0.127
Sex	14 0.699 4 (60.0)

Chi-square analysis was conducted to assess each factor and the passing rate.

## Discussion

### Knowledge among primary care physicians

In general, the medical officers in Seremban District performed well in the knowledge test with a high passing rate. This is in contrast with other reports^6,15^ showing a lower knowledge level among primary care physicians. Our study findings are similar to the findings of studies comparing the knowledge of doctors regarding MAFLD^5,7^ whereby most doctors were able to correctly identify the major risk factors associated with MAFLD and would choose to manage patients with weight reduction and low- caloric diets. We think that the good knowledge level among our study population is surprising and needs to be verified by further studies.

Possible reasons for such a good knowledge level include the large number of patients seen with chronic diseases. There is also a possibility of response bias due to the online nature of the study.

There were discrepancies in the responses to the knowledge items such as the diagnosis of MAFLD in lean patients (43.3%), interpretation of LFT results in MAFLD (45.8%), selection of the gold standard for diagnosing MAFLD (40%) and recommendation of silymarin in managing MAFLD (12.5%). This may be due to the lack of local guidelines and training available to the medical officers regarding MAFLD. The lack of local guidelines on MAFLD was also reported as the cause of poor knowledge among doctors in other studies.^7,17^ These knowledge gaps indicate an ongoing misconception in the local setting and are areas that should be addressed in future educational activities.

In our study, 70% of the practitioners believed that milk thistle (silymarin) can be used to reduce the progression of MAFLD. In an RCT of silymarin treatment for biopsy-proven NASH, a larger proportion of patients in the silymarin group had fibrosis improvement than that in the placebo group. Studies have shown mixed evidence for silymarin use in MAFLD. It may reduce liver fibrosis, but this remains to be confirmed in a larger trial. However, silymarin has not been shown to reduce the NAFLD activity score.^18^

### Practice among primary care physicians

More than 50% of the primary care physicians in this study were not familiar with non-invasive scoring in assessing patients with MAFLD. This may be explained by the lack of awareness among medical officers in using non-invasive scoring. Another factor could be that liver- related diseases such as chronic hepatitis B and C are mostly managed by FMSs. Hence, noninvasive scoring could be more familiar to FMSs than to medical officers. Primary care physicians are rightly placed in the detection of early liver disease, they are the first points of contact for most patients. As such, these physicians should be equipped with the latest updates in the management of MAFLD.

Vitamin E 800 IU is found to significantly improve biopsy-proven NASH and can be used for weight loss in MAFLD.^15^ Most primary care physicians are unaware of this approach similar to our study findings (only 32.5% chose this treatment, while 35% were not sure). Some may have concerns regarding prostate cancer and coronary artery disease with vitamin E supplementation.^15^

In our study, most participants (45.5%) were not sure whether to recommend pioglitazone in managing MAFLD. According to evidence, pioglitazone is recommended (off-label) for biopsy-proven NASH.^22^ Some practitioners may have concerns, as it is not recommended in patients with DM because it increases the risk of weight gain and heart failure.^19-21^

Many medical officers were unable to confidently determine whether changes in dietary composition are involved in managing MAFLD, which is surprising since caloric restriction is the mainstay management of MAFLD.^15^ This knowledge gap shows that dietitian referral may be necessary, as medical officers lack confidence in counselling patients.

When investigations are suggestive of MAFLD, our participants had a mixed response regarding referral to a gastroenterologist. A possible explanation is that primary care physicians may believe that the mainstay management of MAFLD is lifestyle modification, which is under the jurisdiction of primary care physicians. Furthermore, the lack of knowledge and confidence regarding referral indications could be another cause.^15,16^

We found that the medical officers undergoing postgraduate training had a somewhat higher knowledge score than their counterparts, but no difference in the passing rate was noted between them. While postgraduate training in family medicine is now generally regarded as essential, its impact on MAFLD knowledge appeared to be small. We recommend further training to improve the management of MAFLD in primary care settings. Suggestions include having CME on MAFLD, formulating a local clinical practice guideline (CPG) on MAFLD and encouraging medical officers to pursue postgraduate training.

### Study strengths and limitations

The strength of our study is the focus on the knowledge and practice of primary care doctors who are well placed to screen for MAFLD. In addition, the overwhelming response rate (84.5%) showed that the participants had considerable interest in our study.

One limitation of the study is that the findings cannot be extrapolated nationwide, as the study was conducted in only one District in Negeri Sembilan and excluded primary care physicians in private settings and universities. There are also concerns of selection bias due to the use of convenience sampling and response bias due to the online nature of the questionnaire.

## Conclusion

Our study showed that most primary care doctors in Seremban District have good knowledge in diagnosing MAFLD, which is reassuring. There are some gaps in knowledge such as diet recommendations and the use of silymarin, vitamin E and pioglitazone, which may require further education. However, the findings cannot be generalisable nationwide, as the study was conducted only in Seremban District. We suggest that a nationwide study be conducted to determine the overall knowledge level of primary care doctors in Malaysia.

This study highlights the importance of having a local CPG to guide primary care doctors in the management of MAFLD. We suggest having regular CME on MAFLD, formulating a local CPG on MAFLD and encouraging medical officers to pursue postgraduate training.

## Data sharing statement

The participants’ identity was kept anonymous since they did not need to provide any personal details (i.e.name, IC or phone number). The completed questionnaires were stored in a password-protected computer and were handled only by the researchers. All information gathered was confidential and used for research purposes only. Qualified monitors and auditors as well as governmental or regulatory authorities may inspect the study data, where appropriate and necessary. The results of this study will be disseminated or published from pooled data and analysis.

**How does this paper make a difference in general practice?**

- Primary care physicians in Seremban District have good knowledge on metabolic-associated fatty liver disease (MAFLD).
- However, the following gaps in their practice are noted: lack of familiarity with non-invasive laboratory tests and medications and specific diet modifications for the management of MAFLD.
- We suggest having regular CME on MAFLD, encouraging medical officers to pursue postgraduate training and formulating formal clinical practice guidelines to guide management of MAFLD, especially in primary care settings.
